# A first assessment of *Fraxinus excelsior* (common ash) susceptibility to *Hymenoscyphus fraxineus* (ash dieback) throughout the British Isles

**DOI:** 10.1038/s41598-017-16706-6

**Published:** 2017-11-29

**Authors:** Jonathan J. Stocks, Richard J. A. Buggs, Steve J. Lee

**Affiliations:** 1 School of Biological and Chemical Sciences, Queen Mary University of London, London, E1 4NS UK; 2 Royal Botanic Gardens, Kew, Richmond, Surrey, TW9 3AE UK; 3 Forest Research, Northern Research Station, Roslin Midlothian, EH25 9SY UK

## Abstract

Ash dieback (ADB), caused by *Hymenoscyphus fraxineus*, has severely damaged a large proportion of ash trees (*Fraxinus excelsior*) in continental Europe. We have little damage data for the British Isles where the disease was found only five years ago in the Southeast, and is still spreading. A large-scale screening trial to evaluate ADB damage to provenances of *F. excelsior* sourced from throughout the British Isles was planted in 2013 in the southeast of England. In 2016, we scored trees by their level of ADB damage observed in field at the two worst affected (based on assessments in 2015) of the 14 sites. Significant differences were found in average ADB damage among planting sites and seed source provenances. Trees from certain provenances in Scotland were the least damaged by ADB, whereas trees from Wales and Southeast England were the most badly damaged in both trial sites. Thus the levels of ADB damage currently seen in ash populations in Southeast England may not be an accurate predictor of the damage expected in future throughout the British Isles. Given all provenances contained some healthy trees, a breeding programme to produce genetically variable native ash tree populations with lower ADB susceptibility may be feasible.

## Introduction

Ash dieback has rapidly spread through Europe in the last two decades^1,2^. It is caused by the fungus *Hymenoscyphus fraxineus* (T. Kowalski) Baral, Queloz & Hosoya^3–5^, which is native to East Asia. In Europe it is aggressive and invasive^5–8^. It spreads via wind borne spores^9^, and movement of leaves and soil^10^ and was detected in the UK for the first time in native woodlands in 2012^11^.

Many studies have evaluated the damage caused to ash trees in Europe by *H. fraxineus*. For example, young trees planted in Northwest Germany had mortality of 73% five years after planting^12^ (as cited in Enderle *et al*.^13^). Permanent woodland monitoring plots in Lithuania showed 61.1% mortality from 2008–2015 and only 1.9% trees symptomless in 2015^14^. In Norway, an average mortality rate of 57.2% was observed in 2016 in ash dominated forest stands^15^. Several studies show that tree age is a significant factor in the severity of the infection. In the Norwegian assessment of plots in natural forest stands, 80% of the young trees were dead in 2016, whereas for the dominant trees, this number only just surpassed 20%^15^. Surveys carried out in France and Belgium found that mortality was much higher in younger stands (35% 5–6 years after infection) than in older stands (3.2% 8–9 years after infection)^16^.

Susceptibility to *H. fraxineus* has a strong genetic component. Susceptibility to ADB was shown to differ among source provenances in Lithuania and Germany^17,18^. In Denmark, clonal trials established at two sites in 1998 showed highly significant variation in susceptibility among clones with coefficients of broad-sense heritability from 0.40 to 0.49^19,20^. Similar results were found by Lobo *et al*. in Denmark^21,22^, Stener in Sweden^23^, Pliura *et al*. in Lithuania^17,24^, Muñoz *et al*. in France^25^ and Enderle *et al*. in Germany^26^.

In the British Isles, *H. fraxineus* inoculum levels and damage are currently worst in the Southeast of England. The fungus is still spreading though the North and West of Great Britain, and in Ireland. For policy makers to manage the effects of the epidemic, we need accurate predictions of the severity of future damage. In the British Isles this is difficult as ash populations in the North and West appear to be derived from a Pleistocene refugium in Iberia, whereas populations in the south and east are more closely related to trees in France, Germany and the Low Countries^27–30^. Sutherland *et al*. found two chloroplast haplotypes that were widespread in Great Britain, and three that were found only in Scotland, but not the Northwest of Scotland, which had a more common British haplotype^31^. This means the future impact of ADB may vary for different locations in the British Isles. Predictions using transcriptome markers^32^ have suggested that ash trees in the North and West of Great Britain may be less damaged by ash dieback than Danish populations^30^.

Here, we provide the first in-field assessments of the damage caused by ash dieback to genotypes of ash sourced from locations across the British Isles. Our results are based on screening trials of ash saplings from British, Irish and continental provenances set up in 2013 in the Southeast of England. We report detailed assessments of ADB damage from the autumn of 2016, after three and a half year’s exposure to natural *H. fraxineus* inoculum.

## Methods

### Trial design

This study is based on a Forest Research screening experiment planted in spring 2013, comprising 48 hectares of trials on 14 sites in Southeast England. Saplings were sourced from five nurseries in the UK (Supporting Information Table S3) and each site was planted with trees grown from seed sourced from up to 15 different provenances. These were 10 British native seed zones (NSZ 106, NSZ 107, NSZ 109, NSZ 201, NSZ 204, NSZ 302, NSZ 303, NSZ 304, NSZ 403, NSZ 405), Germany (DEU), France (FRA), Ireland (CLARE and IRL DON), and a Breeding Seedling Orchard (BSO) planted by Future Trees Trust (FTT) comprised of half-sibling families from “plus” trees across Britain (for more details see Supporting Information Table S1). Each provenance had two to six complete replications per site, in 16 m × 16 m blocks of 256 trees, randomly distributed (Supporting Information Table S2). All saplings were inspected, and in some cases tested, to ensure they were disease free at the time of planting. In September 2014, establishment of the saplings in all sites was surveyed by Forest Research. In autumn 2015, all sites were surveyed by Forest Research with each tree scored as 1 – dead or missing, 2 – alive and infected and 3 – alive and healthy (Forest Research, unpublished data).

### Detailed Phenotyping

In autumn 2016 we scored the ash dieback symptoms of 28,160 trees at the two trial sites that had showed highest damage in 2015, which were located near Norwich in Norfolk (Site 16) and near Royal Tunbridge Wells in East Sussex (Site 35). We considered that these sites had the highest inoculum pressure, and would be the most reliable sites for gaining a preliminary understanding of the susceptibility of different provenances to ADB, as fewer trees would be likely to have simply avoided infection by chance. We used the methodology of Pliura *et al*. to score the damage phenotypes^17^ of the trees in these two sites. This method uses a scale of 1–7 (Supporting Information Fig. 1S, Supporting Information Fig. 2S) as follows: 1 – missing tree; 2 – dead, dry tree without significant development; 3 – dry tree that produced shoots before dying; 4 – heavily damaged living tree with dead main stem and resprouted stems with highly visible leaf and stem ADB lesions; 5 – moderate damage with dry leading shoot and once or repeatedly resprouted leading shoot with highly visible leaf and stem ADB lesions; 6 – limited damage with multiple brown dry or wilted leaves or peripheral shoot or/and brown lesions on stem or branches; 7 – healthy tree with minimal signs of damage only on leaves (brown, dry or wilted leaves). We later introduced a category of zero for trees that were missing both in autumn 2016 and autumn 2014: these trees were subtracted from score one.

We analysed the damage scores using a general linear model in a factorial ANOVA with score as the dependent variable and site and seed source provenances as categorical predictors (factors). For this analysis, seed source provenances that did not have an equal amount of replication (NSZ 403 has only three blocks at Site 16) and seed source provenances that are not present at both sites (DEU, FRA and CLARE only present at Site 16) were excluded. Score zero and one (missing trees) were excluded from our initial analyses since trees in both categories most likely died for reasons other than ADB. In case score one trees has in fact died from ash dieback, we repeated the analyses including score one trees and only excluding score zero trees. We then analysed each site separately, including all seed source provenances present at that site and conducting a post-hoc pairwise comparison (Tukey’s test) to find significant differences between provenances; this was done twice, once with score one and zero trees excluded, and then with only score zero trees excluded.

### Data availability

The dataset generated and analysed during the current study are available as Supplementary Data.

## Results

**Figure 1:**
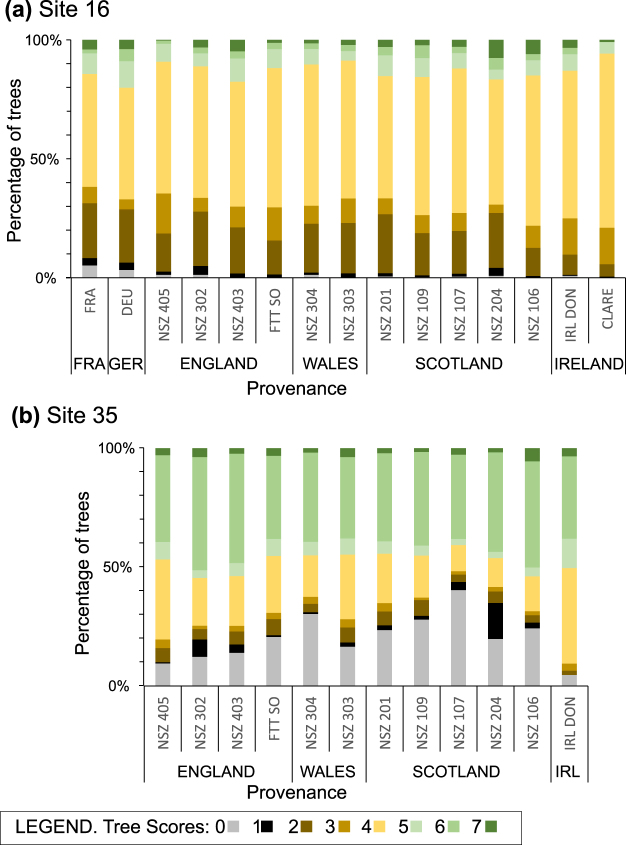
Percentage of trees in each damage score for all provenances planted at: (**a**) Site 16; (**b**) Site 35. The total number of trees from each provenance planted at each site can be found in Supporting Information Table S1.

The percentages of trees in each damage class, site and from each provenance are shown in Fig. 1a,b. Site 35 had a higher percentage (20.3% Score zero and 3.3% Score one) of missing trees than Site 16 (1.1% Score zero and 1.7% Score one) for all provenances (Fig. 1b, Supporting Information Fig. S3). We are convinced that this was due to poor establishment at Site 35 and not caused by ash dieback for three reasons: (1) The majority of missing trees had disappeared by autumn 2014, (2) because the trees that did establish well at Site 35 were less badly affected by ash dieback in 2016 than the trees at Site 16 (see below), suggesting higher inoculum pressures at Site 16, and (3) the missing trees at Site 35 were spatially clustered in a wide strip suggestive of a soil condition problem, such as poor drainage, rather than the spread of wind-borne spores. We therefore excluded Score zero trees from our analyses of ash dieback damage at both sites. For Score one, where trees were present in 2014 but missing by 2016, we could not be sure if they had died from ADB or not, so we analysed the data twice, one including and one excluding them.

The two sites showed significant (p ≤ 0.001) differences between them in level of ADB damage (Table 1). The average score (excluding Score zero and one trees), for Site 35 was 5.05 (S.D. = 1.28) and for Site 16 was 3.76 (S.D. = 1.12). When only Score zero trees were excluded, in case Score one trees had in fact died from ADB, the same pattern was found with the average score for Site 35 being 4.89 (S.D. = 1.49) and for Site 16 being 3.72 (S.D. = 1.17). This pattern suggests that site 35 has experienced a lower inoculum pressure than site 16, perhaps partly due to a lower density of established trees, and is therefore at an earlier stage of infection. Average mortality that can be definitely attributed to ash dieback (Scores two and three) at Site 16 was 27% and at Site 35 was 7%, with an average across both sites of 18%. Average percentage of healthy trees (Score seven) at Site 16 was 3.1% and at Site 35 was 2.9%.

**Table 1:** Effect of site and provenance on tree damage scores in GLM ANOVA tables: (a) excluding Scores 0 and 1 trees; (b) excluding Score 0 trees.

(a) Effect	df	Adj. MS	F
***Factorial***			
Site	1	8466.3	6042.8*
Provenance NSZ	10	34.9	24.9*
Provenance NSZ * Site	10	14.0	10.0*
Error	20,077	1.4	
Total	20,098		
**(b) Effect**	**df**	**Adj. MS**	**F**
***Factorial***			
Site	1	7077.7	4121.1*
Provenance NSZ	10	35.7	20.8*
Provenance NSZ * Site	10	19.5	11.4*
Error	20,636	1.7	
Total	20,657		

Site and seed source provenance were included as factors and provenances not present at both sites and in all blocks (DEU, FRA, CLARE, and NSZ 403) were excluded. *p ≤ 0.001.

**Figure 2:**
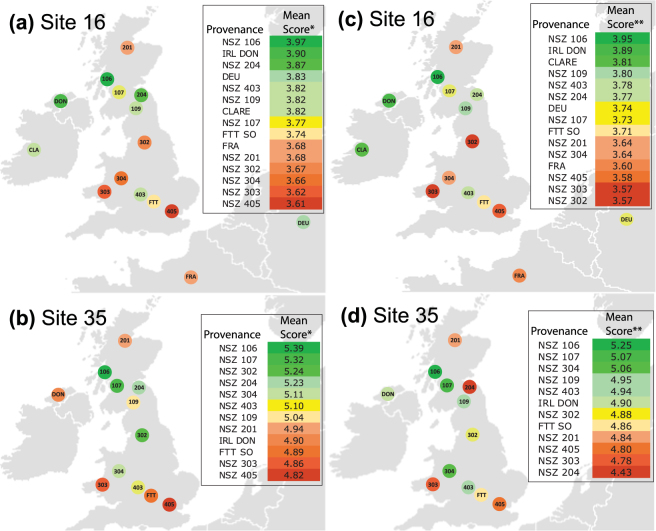
Maps of provenance source locations showing average tree score at trial sites 16 and 35. (**a**) Mean scores for Site 16 (*excluding Score 0 and 1); (**b**) Mean scores for Site 35 (*excluding Score 0 and 1); (**c**) Mean scores for Site 16 (**excluding Score 0); (**d**) Mean scores for Site 35 (**excluding Score 0).

Provenance was a significant factor for ash dieback damage when differences between sites were accounted for (Table 1). This was true whether or not Score one trees were included in the analysis. Pairwise comparisons of provenance scores are shown in Table 2, where both Score zero and score one trees are excluded. The Scottish provenance NSZ 106 had the highest average score at both sites, being 3.97 at Site 16 (Table 2a & Fig. 2a,c) and 5.39 at Site 35 (Table 2b and Fig. 2b,d). Mortality figures for NSZ 106 are amongst the lowest for all provenances (Table 2a,b and Table 3a,b). Overall, NSZ106 is significantly different (p ≤ 0.05) to eight of the fifteen provenances at Site 16 (Table 2a) and eight of the twelve provenances at Site 35 (Table 2b). The British southeastern provenance NSZ 405 had the lowest average score at both sites (Table 2a,b & Fig. 2a,b) with the highest mortality (Table 2a,b) for all provenances. The BSO FTT provenance, which is comprised of superior trees for commercial forestry, was found to have low (Site 35) to intermediate (Site 16) average scores in this study (Table 2a,b). Performance analysis for FRA, DEU and CLARE can only be made at Site 16, as they were not planted at Site 35. Interestingly, CLARE has very few trees at the upper end of the scoring scheme (0% Score six and 1% Score seven) (Fig. 1a), a large percentage of highly damaged trees (73% Score four) but also lower mortality (Scores two and three) at 21% (Table 2a). The German provenance (DEU) has the second highest percentage of Score six (limited damage) trees and performed well overall, with an average score of 3.83 at Site 16 (Table 2a).

**Table 2:** Differences in tree damage scores among provenances, with score 0 and 1 trees excluded, at (a) Site 16 and (b) Site 35.

(a)	NSZ	Score (%)		Count	$\bar{{\rm{x}}}$	σ^2^	*o*	*n*	*m*	*l*	*k*	*j*	*i*	*h*	*g*	*f*	*e*	*d*	*c*	*b*	*a*
		2-3	7																		
a	NSZ 106	21	6.0	1,016	3.97	1.281									|	|	|	|	|	|	|
b	IRL DON	24	3.3	1,011	3.90	0.973							|	|	|	|	|	|	|	|	|
c	NSZ 204	27	7.6	981	3.87	1.945							|	|	|	|	|	|	|	|	|
d	DEU	27	3.9	958	3.83	1.686			|	|	|	|	|	|	|	|	|	|	|	|	|
e	NSZ 403	28	4.8	754	3.82	1.513			|	|	|	|	|	|	|	|	|	|	|	|	|
f	NSZ 109	25	2.2	1,013	3.82	1.270			|	|	|	|	|	|	|	|	|	|	|	|	|
g	CLARE	21	0.9	1,019	3.82	0.464			|	|	|	|	|	|	|	|	|	|	|	|	|
h	NSZ 107	26	2.9	1,006	3.77	1.196	|	|	|	|	|	|	|	|	|	|	|	|	|	|	
i	FTT SO	28	1.3	1,010	3.74	0.961	|	|	|	|	|	|	|	|	|	|	|	|	|	|	
j	FRA	30	4.0	939	3.68	1.542	|	|	|	|	|	|	|	|	|	|	|	|			
k	NSZ 201	31	3.0	1,004	3.68	1.488	|	|	|	|	|	|	|	|	|	|	|	|			
l	NSZ 302	29	3.2	973	3.67	1.380	|	|	|	|	|	|	|	|	|	|	|	|			
m	NSZ 304	28	1.5	1,001	3.66	1.102	|	|	|	|	|	|	|	|	|	|	|	|			
n	NSZ 303	31	2.1	1,005	3.62	1.168	|	|	|	|	|	|	|	|							
o	NSZ 405	33	0.3	997	3.61	0.842	|	|	|	|	|	|	|	|							
**(b)**	**NSZ**	**Score (%)**		**Count**	$\bar{{\bf{x}}}$	**σ** ^**2**^	***l***	***k***	***j***	***i***	***h***	***g***	***f***	***e***	***d***	***c***	***b***	***a***			
		**2-3**	**7**																		
a	NSZ 106	5	5.5	798	5.39	1.437									|	|	|	|			
b	NSZ 107	5	2.8	612	5.32	1.522							|	|	|	|	|	|			
c	NSZ 302	6	3.8	875	5.24	1.529						|	|	|	|	|	|	|			
d	NSZ 204	7	1.7	708	5.23	1.665						|	|	|	|	|	|	|			
e	NSZ 304	6	1.8	750	5.11	1.521					|	|	|	|	|	|	|				
f	NSZ 403	8	2.3	898	5.10	1.621					|	|	|	|	|	|	|				
g	NSZ 109	8	1.6	767	5.04	1.801		|	|	|	|	|	|	|	|	|					
h	NSZ 201	9	2.1	811	4.94	1.786	|	|	|	|	|	|	|	|							
i	IRL DON	5	3.4	1,037	4.90	1.207	|	|	|	|	|	|									
j	FTT SO	10	3.2	856	4.89	1.816	|	|	|	|	|	|									
k	NSZ 303	10	3.8	889	4.86	1.784	|	|	|	|	|	|									
l	NSZ 405	10	2.9	979	4.82	1.631	|	|	|	|	|										

For each provenance we show: percentage of trees with score 2 and 3, percentage of healthy (score 7) trees, total number of trees, mean and variance of score, and Tukey pairwise comparison of provenances (where | signifies no significant difference at the 0.05 level).

**Table 3:** Differences in tree damage scores among provenances, with score 0 trees excluded, at (a) Site 16 and (b) Site 35.

(a)	NSZ	Score (%)		Count	$\bar{{\rm{x}}}$ ^(1)^	σ^2^	*o*	*n*	*m*	*l*	*k*	*j*	*i*	*h*	*g*	*f*	*e*	*d*	*c*	*b*	*a*
		2–3	7																		
a	NSZ 106	21.1	6.0	1,022	3.95	1.324											|	|	|	|	|
b	IRL DON	23.7	3.3	1,015	3.89	1.002								|	|	|	|	|	|	|	|
c	CLARE	20.6	0.9	1,023	3.81	0.493					|	|	|	|	|	|	|	|	|	|	|
d	NSZ 109	25.3	2.2	1,021	3.80	1.322					|	|	|	|	|	|	|	|	|	|	|
e	NSZ 403	28.1	4.8	766	3.78	1.613				|	|	|	|	|	|	|	|	|	|	|	|
f	NSZ 204	26.6	7.6	1,015	3.77	2.146				|	|	|	|	|	|	|	|	|	|	|	
g	DEU	26.6	3.9	990	3.74	1.882	|	|	|	|	|	|	|	|	|	|	|	|	|	|	
h	NSZ 107	25.5	2.9	1,019	3.73	1.277	|	|	|	|	|	|	|	|	|	|	|	|	|	|	
i	FTT SO	28.2	1.3	1,022	3.71	1.037	|	|	|	|	|	|	|	|	|	|	|	|	|		
j	NSZ 201	31.4	3.0	1,018	3.64	1.565	|	|	|	|	|	|	|	|	|	|	|	|	|		
k	NSZ 304	28.0	1.5	1,011	3.64	1.160	|	|	|	|	|	|	|	|	|	|	|	|	|		
l	FRA	29.9	4.0	971	3.60	1.721	|	|	|	|	|	|	|	|	|	|	|				
m	NSZ 405	32.8	0.3	1,011	3.58	0.924	|	|	|	|	|	|	|	|	|						
n	NSZ 303	31.4	2.1	1,024	3.57	1.271	|	|	|	|	|	|	|	|	|						
o	NSZ 302	28.6	3.2	1,011	3.57	1.586	|	|	|	|	|	|	|	|	|						
**(b)**	**NSZ**	**Score (%)**		**Count**	$\bar{{\bf{x}}}$ ^**(1)**^	**σ** ^**2**^	***l***	***k***	***j***	***i***	***h***	***g***	***f***	***e***	***d***	***c***	***b***	***a***			
		**2–3**	**7**																		
a	NSZ 106	4.8	5.5	824	5.25	1.980										|	|	|			
b	NSZ 107	4.5	2.8	649	5.07	2.438				|	|	|	|	|	|	|	|	|			
c	NSZ 304	6.4	1.8	758	5.06	1.682				|	|	|	|	|	|	|	|	|			
d	NSZ 109	7.6	1.6	784	4.95	2.109		|	|	|	|	|	|	|	|	|	|				
e	NSZ 403	7.8	2.3	936	4.94	2.212		|	|	|	|	|	|	|	|	|	|				
f	IRL DON	4.7	3.4	1,037	4.90	1.207		|	|	|	|	|	|	|	|	|	|				
g	NSZ 302	5.8	3.8	955	4.88	2.781		|	|	|	|	|	|	|	|	|	|				
h	FTT SO	9.5	3.2	864	4.86	1.938		|	|	|	|	|	|	|	|	|	|				
i	NSZ 201	9.4	2.1	832	4.84	2.124		|	|	|	|	|	|	|	|	|	|				
j	NSZ 405	9.6	2.9	985	4.80	1.709		|	|	|	|	|	|	|	|						
k	NSZ 303	9.7	3.8	908	4.78	2.052		|	|	|	|	|	|	|	|						
l	NSZ 204	6.7	1.7	873	4.43	4.096	|														

For each provenance we show: percentage of trees with score 2 and 3, percentage of healthy (score 7) trees, total number of trees, mean and variance of score, and Tukey pairwise comparison of provenances (where|signifies no significant difference at the 0.05 level). ^(1)^NSZ not followed by | are significantly different (Tukey pairwise 5%); Excluded missing trees.

When Score one trees were included in the analysis, in case they had died from ADB, the overall pattern of results was similar for Site 16. At Site 35, two provenances, NSZ 204 and NSZ 302 decreased in their average score (18% and 7% respectively), altering their ranking with respect to other provenances at the site (Table 3 and Fig. 2d).

## Discussion

We found significant differences among provenances of ash trees in their susceptibility to ash dieback in the British Isles. Trees from the middle of Scotland appeared least susceptible and interestingly, these areas were also found, by a previous study, to contain rare chloroplast haplotypes^31^. Sutherland *et al*. suggested that these were sourced from a separate glacial refugium to other ash populations in Great Britain^31^.

The most southeasterly British ash provenance in our experiment was consistently amongst the most damaged. Trees from this provenance sampled in Sutherland *et al*. had the same chloroplast haplotype as most of Great Britain^31^, but the study of Heuertz *et al*. showed this provenance to also contain haplotypes present in France, Switzerland and Italy^27^. Thus this provenance may be more closely related to the French ash trees that have also shown high mortality in this study. Our results suggest that current high damage due to ADB in the Southeast of England may be greater than what we might expect to see throughout the rest of the British Isles in the coming years as ADB continues to spread. In particular, parts of Scotland may be less badly affected.

Our results suggest that a few ash trees with low susceptibility to ADB are present in most provenances from across the British Isles. Similar results have been found in Europe^15,19,22,23,33^. It should be remembered that this study represents just two of the 14 sites planted in 2013 and the trees are only four years old, but these early findings suggest that the prospects for selective breeding for lower susceptibility to ADB in a variety of native provenances are good. The suite of Forest Research trial sites will provide valuable starting materials for such breeding programmes in the near future.

## Electronic supplementary material

Supplementary Information

Dataset 1

## References

[1] Timmermann V, Børja I, Hietala AM, Kirisits T, Solheim H (2011). Ash dieback: pathogen spread and diurnal patterns of ascospore dispersal, with special emphasis on Norway. Bull. OEPP.

[2] Pautasso M, Gregor A, Queloz V, Holdenrieder O (2013). European ash (*Fraxinus excelsior*) dieback – a conservation biology challenge. Biol. Conserv..

[3] Kowalski T (2006). *Chalara fraxinea* sp. nov. associated with dieback of ash (*Fraxinus excelsior*) in Poland. For. Pathol..

[4] Baral HO, Queloz V, Hosoya T (2014). *Hymenoscyphus fraxineus*, the correct scientific name for the fungus causing ash dieback in Europe. IMA Fungus.

[5] Gross A, Holdenrieder O, Pautasso M, Queloz V, Sieber TN (2014). *Hymenoscyphus pseudoalbidus*, the causal agent of European ash dieback. Mol. Plant Pathol..

[6] Zhao Y, Hosoya T, Baral HO, Hosaka K, Kakishima M (2012). *Hymenoscyphus pseudoalbidus*, the correct name for *Lambertella albida* reported from Japan. Mycotaxon.

[7] McKinney LV (2014). The ash dieback crisis: genetic variation in resistance can prove a long-term solution. Plant Pathol..

[8] Kräutler K, Treitler R, Kirisits T (2015). *Hymenoscyphus fraxineus* can directly infect intact current-year shoots of *Fraxinus excelsior* and artificially exposed leaf scars. For. Pathol..

[9] Kowalski T, Holdenrieder O (2009). Pathogenicity of *Chalara fraxinea*. For. Pathol..

[10] Fones HN, Mardon C, Gurr SJ (2016). A role for the asexual spores in infection of *Fraxinus excelsior* by the ash-dieback fungus *Hymenoscyphus fraxineus*. Sci. Rep..

[11] British Ecological Society. *Ecology and Policy Blog*, http://www.britishecologicalsociety.org/first-occurrence-of-ash-dieback-in-britain/ (2012).

[12] Langer, G., Harriehausen, U., Bressem, U. Eschentriebsterben und Folgeerscheinungen [Ash dieback and its consequences]. *AFZ/Der Wald* **70**(**20**), 22–28, (in German) (2015).

[13] Enderle, R. *et al*. Ash dieback in Germany: research on disease development, resistance and management options in *Dieback of European Ash (Fraxinus spp.): Consequences and Guidelines for* Sustainable *Management* (ed. Vasaitis, R., Enderle, R.) 89–105 (Swedish University of Agricultural Sciences, 2017).

[14] Pliura, A. *et al*. Ash dieback in Lithuania: disease history, research on impact and genetic variation in disease resistance, tree breeding and options for forest management in *Dieback of European Ash (Fraxinus spp.): Consequences and Guidelines for* Sustainable *Management* (eds Vasaitis, R., Enderle, R.) 150–165 (Swedish University of Agricultural Sciences, 2017).

[15] Timmermann V, Nagy NE, Hietala AM, Børja I, Solheim H (2017). Progression of Ash Dieback in Norway Related to TreeAge, Disease History and Regional Aspects. Balt. For..

[16] Marçais B (2017). Estimation of Ash Mortality Induced by *Hymenoscyphus fraxineus* in France and Belgium. Balt. For..

[17] Pliura A, Lygis V, Suchockas V, Bartkevicius E (2011). Performance of twenty-four European *Fraxinus excelsior* populations in three Lithuanian progeny trials with a special emphasis on resistance to *Chalara fraxinea*. Balt. For..

[18] Metzler B, Enderle R, Karopka M, Topfner K, Aldinger E (2012). Entwicklung des Eschentriebsterbens in einem Herkunftsversuch an verschiedenen Standorten in Suddeutschland. Ger. J. For. Res..

[19] McKinney LV, Nielsen LR, Hansen JK, Kjær ED (2011). Presence of natural genetic resistance in *Fraxinus excelsior* (Oleraceae) to *Chalara fraxinea* (Ascomycota): an emerging infectious disease. Heredity.

[20] McKinney LV, Thomsen IM, Kjær ED, Nielsen LR (2012). Genetic resistance to *Hymenoscyphus pseudoalbidus* limits fungal growth and symptom occurrence in *Fraxinus excelsior*. For. Pathol..

[21] Lobo A, Hansen JK, McKinney LV, Nielsen LR, Kjær ED (2014). Genetic variation in dieback resistance: growth and survival of *Fraxinus excelsior* under the influence of *Hymenoscyphus pseudoalbidus*. Scand. J. For. Res..

[22] Lobo A, McKinney LV, Hansen JK, Kjær ED, Nielsen LR (2015). Genetic variation in dieback resistance in *Fraxinus excelsior* confirmed by progeny inoculation assay. For. Pathol..

[23] Stener LG (2013). Clonal differences in susceptibility to the dieback of *Fraxinus excelsior* in southern Sweden. Scan. J. For. Res..

[24] Pliura A, Marèiulynienë D, Bakys R, Suchockas V (2014). Dynamics of genetic resistance to *Hymenoscyphus pseudoalbidus* in juvenile *Fraxinus excelsior* clones. Balt. For..

[25] Muñoz F, Marçais B, Dufour J, Dowkiw A (2016). Rising out of the ashes: additive genetic variation for susceptibility to *Hymenoscyphus fraxineus* in *Fraxinus excelsior*. Phytopathology.

[26] Enderle R, Nakou A, Thomas K, Metzler B (2015). Susceptibility of autochthonous German *Fraxinus excelsior* clones to *Hymenoscyphus pseudoalbidus* is genetically determined. Ann. For. Sci..

[27] Heuertz M (2004). Chloroplast DNA variation and postglacial recolonization of common ash (*Fraxinus excelsior* L.) in Europe. Mol. Ecol..

[28] Heuertz M, Hausman JF, Hardy OJ (2004). Nuclear Microsatellites reveal contrasting patterns of genetic structure between western and south eastern European populations of the common ash (*Fraxinus excelsior* L.). Evolution.

[29] Heuertz M (2006). Chloroplast DNA phylogeography of European ashes, *Fraxinus* sp. (Oleaceae): roles of hybridization and life history traits. Mol. Ecol..

[30] Sollars ESA (2017). Genome sequence and genetic diversity of European ash trees. Nature.

[31] Sutherland BG (2010). Molecular biodiversity and population structure in common ash (*Fraxinus excelsior* L.) in Britain: implications for conservation. Mol. Ecol..

[32] Harper AL (2016). Molecular markers for tolerance of European ash (*Fraxinus excelsior*) to dieback disease identified using Associative Transcriptomics. Sci. Rep..

[33] Kjær ED, McKinney LV, Nielsen LR, Hansen LN, Hansen JK (2012). Adaptive potential of ash (*Fraxinus excelsior*) populations against the novel emerging pathogen *Hymenoscyphus pseudoalbidus*. Evol. Appl..

