# Supplementary material for: A first assessment of Fraxinus excelsior (common ash) susceptibility to Hymenoscyphus fraxineus (ash dieback) throughout the British Isles
Source: Sci Rep. 2017 Nov 29;7:16546. doi: 10.1038/s41598-017-16706-6 (PMC5707348; doi:10.1038/s41598-017-16706-6)
Supplement: Supplementary file 1 — Supplementary Information [file 41598_2017_16706_MOESM1_ESM.doc]

## *Scientific Reports* Supporting Information

Article title: **A first assessment of** *Fraxinus excelsior* **(common ash) susceptibility to** *Hymenoscyphus fraxineus* **(ash dieback) throughout the British Isles**

Authors: Jonathan J. Stocks, Richard J. A. Buggs, Steve J. Lee

**Fig. S1** Scoring methodology.

**Fig. S2** Phenotype assessment pictures.

**Fig. S3** Percentage of trees per score and site.

**Table S1** Provenance origin details.

**Table S2** Research design details.

**Table S3** Nursery of origin details.


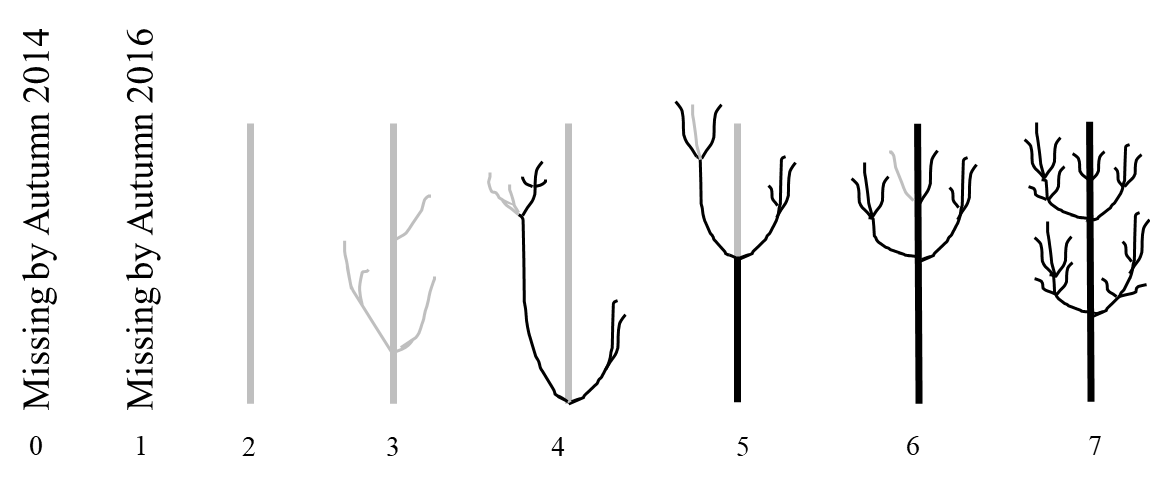


**Fig. S1** Scoring of resistance or tolerance to *Hymenoscyphus fraxineus*. Figure adapted from Pliura *et al.* (2011).

**
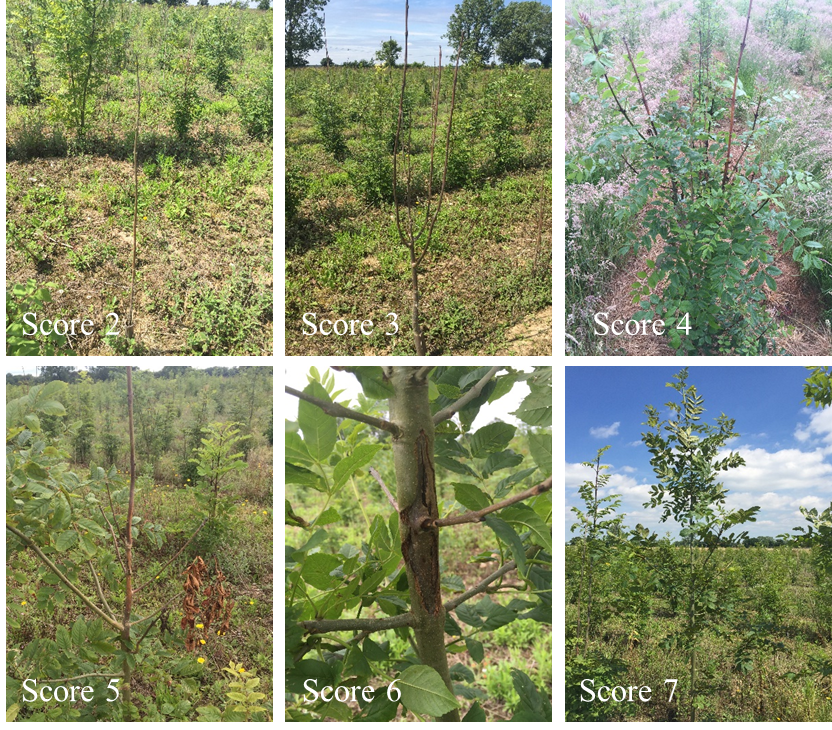
**

**Fig. S2** Phenotype assessment pictures for each score. Photographs by Jonathan Stocks.

**
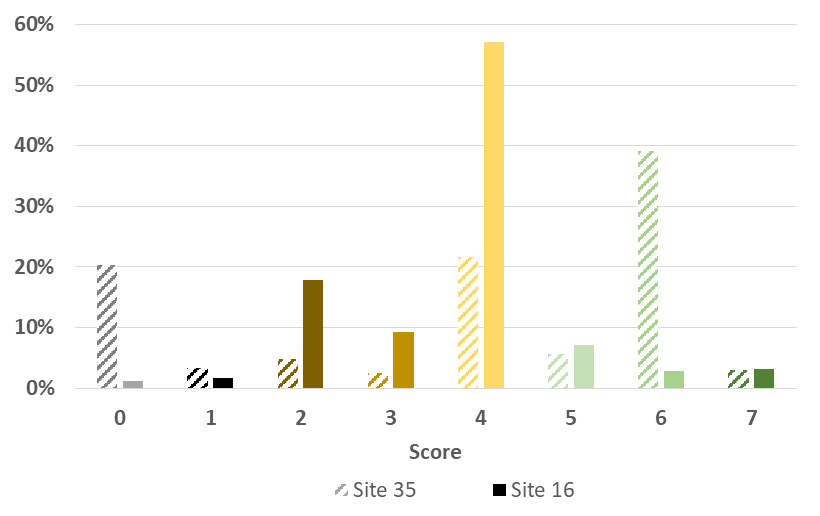
**

**Fig. S3** Percentage of trees for each score at Site 16 and Site 35.

**Table S1 List of provenances and their Native Seed Zones (NSZ), source and type of material details and total number of trees planted at Site 35 and Site 16.**

| **N°** | **Ash Native Seed Zone (NSZ)** | **Type of basic material*** | **Cat.**** | **Country** | **Region of Provenance Code** | **Number of Plants** | | **Seed collection location** |
| --- | --- | --- | --- | --- | --- | --- | --- | --- |
|  |  |  |  |  |  | **Site 35** | **Site 16** |  |
| 1 | NSZ 405 | RP | SI | England | UK40 | 1,088 | 1,024 | Kent |
| 2 | NSZ 403 | RP | SI | England | UK40 | 1,088 | 768 | Shropshire |
| 3 | NSZ 304 | RP | SI | Wales | UK30 | 1,088 | 1,024 | Denbighshire |
| 4 | NSZ 303 | RP | SI | Wales | UK30 | 1,088 | 1,024 | Gwynedd |
| 5 | NSZ 302 | RP | SI | England | UK30 | 1,088 | 1,024 | Cheshire (0-50 m) |
| 6 | NSZ 204 | RP | SI | Scotland | UK20 | 1,088 | 1,024 | Berwick (50-150 m) |
| 7 | NSZ 201 | RP | SI | Scotland | UK20 | 1,088 | 1,024 | Unknown |
| 8 | NSZ 109 | RP | SI | Scotland | UK10 | 1,088 | 1,024 | Attonburn |
| 9 | NSZ 107 | RP | SI | Scotland | UK10 | 1,088 | 1,024 | Unknown |
| 10 | NSZ 106 | RP | SI | Scotland | UK10 | 1,088 | 1,024 | Loch Creran |
| 11 | FTT SO | OR | QU | England | FTT | 1,088 | 1,024 | Earth Trust, Oxfordshire |
| 12 | CLARE | RP | SI | Ireland | IRL | 1,088 | 1,024 | Clare |
| 13 | IRL DON | RP | SI | Ireland | IRL | 1,088 | 1,024 | Donegal |
| 14 | DEU | ST | SE | Germany | DEU | 1,088 | 1,024 | Unknown |
| 15 | FRA | ST | SE | France | FRA | 1,088 | 1,024 | NE France (80 m) |

* RP - Region of Provenance; OR - Seed Orchard; ST - Stand.** SI - Source Identified; QU - Qualified; SE - Selected.

**Table S2 List of all Forest Research trial sites, with location and experimental details.**

| **FR* n°** | **Location** | **Replicates** | **Total n° of Plots** | **Number of Entries** | **Total n° of plants** | **Total planted area (ha)** |
| --- | --- | --- | --- | --- | --- | --- |
| 4 | Suffolk | 6 | 90 | 15 | 24,480 | 4.9 |
| 6 | Norfolk | 3 | 31 | 11 | 7,936 | 1.6 |
| 11 | Norfolk | 4 | 56 | 14 | 14,336 | 2.9 |
| 13 | Suffolk | 3 | 27 | 9 | 6,912 | 1.4 |
| 14 | Lincolnshire | 4 | 60 | 15 | 15,360 | 3.1 |
| 16 | Norfolk | 4 | 60 | 15 | 14,336 | 2.9 |
| 21 | Kent | 4 | 56 | 14 | 8,064 | 1.6 |
| 23 | Norfolk | 3 | 32 | 11 | 8,192 | 1.6 |
| 24 | Hertfordshire | 2 | 26 | 13 | 6,656 | 1.3 |
| 26 | Norfolk | 4 | 60 | 15 | 17,184 | 3.4 |
| 27 | Suffolk | 3 | 24 | 8 | 6,144 | 1.2 |
| 31 | Kent | 4 | 40 | 10 | 5,760 | 1.2 |
| 32 | Kent | 4 | 40 | 10 | 5,760 | 1.2 |
| 35 | East Sussex | 4 | 48 | 12 | 12,288 | 2.5 |
| *Project site number according to Forest Research (FR) | | | |  |  |  |

**Table S3** Average score for each nursery of origin at Site 16, Site 35 and at both sites.

| **Nursery** | **Nursery Location** | **Provenances sourced from nursery** | **Average Damage w/o Score 0 & 1** | | | | **Average Damage incl. Score 1** | | |
| --- | --- | --- | --- | --- | --- | --- | --- | --- | --- |
|  |  |  | **Site 16** | **Site 35** | **Both Sites** | **Site 16** | | **Site 35** | **Both Sites** |
| Cheviot Trees Ltd | Berwick Upon Tweed | NSZ 302 | 3.74 | 5.23 | 4.27 | 3.65 | | 4.67 | 4.03 |
|  |  | NSZ 204 |  |  |  |  |  |  |  |
|  |  | FRA |  |  |  |  |  |  |  |
| Maelor Forest Nurseries Ltd | Shropshire | NSZ 106 | 3.78 | 5.10 | 4.31 | 3.74 | | 5.00 | 4.25 |
|  |  | NSZ 107 |  |  |  |  |  |  |  |
|  |  | NSZ 109 |  |  |  |  |  |  |  |
|  |  | NSZ 201 |  |  |  |  |  |  |  |
|  |  | NSZ 304 |  |  |  |  |  |  |  |
|  |  | FTT SO |  |  |  |  |  |  |  |
|  |  | DEU |  |  |  |  |  |  |  |
| Delamere FC Nurseries | Cheshire | NSZ 303 | 3.71 | 4.98 | 4.35 | 3.66 | | 4.86 | 4.27 |
|  |  | NSZ 403 |  |  |  |  |  |  |  |
| Oakover Nurseries | Kent | NSZ 405 | 3.61 | 4.82 | 4.21 | 3.58 | | 4.80 | 4.18 |
| Coillte |  | CLARE | 3.86 | 4.90 | 4.21 | 3.85 | | 4.90 | 4.20 |
|  |  | IRL DON |  |  |  |  |  |  |  |
